# When a discriminating dose assay is not enough: measuring the intensity of insecticide resistance in malaria vectors

**DOI:** 10.1186/s12936-015-0721-4

**Published:** 2015-05-20

**Authors:** Judit Bagi, Nelson Grisales, Rebecca Corkill, John C Morgan, Sagnon N’Falé, William G Brogdon, Hilary Ranson

**Affiliations:** Liverpool School of Tropical Medicine, Pembroke Place, Liverpool, L3 5QA UK; Centre National de Recherche et de la Formation sur Paludisme, Ouagadougou, 01BP2208 Burkina Faso; Division of Parasitic Diseases and Malaria, Center for Global Health, Centers for Disease Control, Atlanta, GA 30329 USA

## Abstract

**Background:**

Guidelines from the World Health Organization for monitoring insecticide resistance in disease vectors recommend exposing insects to a predetermined discriminating dose of insecticide and recording the percentage mortality in the population. This standardized methodology has been widely adopted for malaria vectors and has provided valuable data on the spread and prevalence of resistance. However, understanding the potential impact of this resistance on malaria control requires a more quantitative measure of the strength or intensity of this resistance.

**Methods:**

Bioassays were adapted to quantify the level of resistance to permethrin in laboratory colonies and field populations of *Anopheles gambiae sensu lato*. WHO susceptibility tube assays were used to produce data on mortality versus exposure time and CDC bottle bioassays were used to generate dose response data sets. A modified version of the CDC bottle bioassay, known as the Resistance Intensity Rapid Diagnostic Test (I-RDT), was also used to measure the knockdown and mortality after exposure to different multipliers of the diagnostic dose. Finally cone bioassays were used to assess mortality after exposure to insecticide treated nets.

**Results:**

The time response assays were simple to perform but not suitable for highly resistant populations. After initial problems with stability of insecticide and bottle washing were resolved, the CDC bottle bioassay provided a reproducible, quantitative measure of resistance but there were challenges performing this under field conditions. The I-RDT was simple to perform and interpret although the end point selected (immediate knockdown versus 24 h mortality) could dramatically affect the interpretation of the data. The utility of the cone bioassays was dependent on net type and thus appropriate controls are needed to interpret the operational significance of these data sets.

**Conclusions:**

Incorporating quantitative measures of resistance strength, and utilizing bioassays with field doses of insecticides, will help interpret the possible impact of resistance on vector control activities. Each method tested had different benefits and challenges and agreement on a common methodology would be beneficial so that data are generated in a standardized format. This type of quantitative data are an important prerequisite to linking resistance strength to epidemiological outcomes.

## Background

Insecticides are a vital part of the malaria vector control tool box. In sub-Saharan Africa, where the vast majority of malaria morbidity and mortality occurs, the World Health Organization (WHO) recommends universal coverage with long-lasting insecticide nets (LLINs) treated with pyrethroids to reduce malaria transmission. Pyrethroids, plus also DDT, organophosphates and carbamates, are also being used in Indoor Residual Spraying (IRS) programmes in many African countries. The scale-up in coverage with LLINs and IRS have imposed a massive selection pressure on the malaria mosquitoes resulting in an escalation in insecticide resistance. This is compounded by the use of the same classes of chemicals in agriculture and, for pyrethroids, also in consumer products such as aerosols and coils [1].

Pyrethroid resistance was first detected in the two major malaria vectors *Anopheles gambiae s.l.* and *Anopheles funestus* in a small number of sites at the end of the last century [2–4]. The 21^st^ century has witnessed a rapid spread of this resistance phenotype across the continent and it is now difficult to find sites in Africa where both vectors remain fully susceptible to pyrethroids [5]. Several different resistance mechanisms have been detected. Single amino acid substitutions at codon 1014 of the pyrethroid target site, the voltage gated sodium channel (known as *kdr* mutations), were the first mechanisms to be molecularly characterized [6, 7]. Further target site mutations have now been reported [8] in addition to potentially more potent metabolic and/or penetration based mechanisms [9, 10].

The vast majority of resistance monitoring in malaria vectors follows WHO protocols, revised in 2013, which recommend the use of susceptibility tube bioassays with papers coated with ‘discriminatory doses’ of insecticide [11]. Data are reported as percentage mortality and a threshold of less than 90 % mortality is used to define resistance (and mortality between 90 and 98 % is defined as suggestive of the presence of resistance). This standardized methodology is useful for tracking the spread of resistance but does not provide information on the strength of this resistance or its impact. The concentration of insecticide used has no relationship to the quantity of insecticide used in field applications but is instead set as twice the concentration required to kill a susceptible strain of the same species. Furthermore, by using prevalence of resistance as the metric, it is not possible to identify regions where resistance is likely to be posing the greatest threat to malaria control. Mosquitoes collected from site A may yield 50 % mortality in a discriminating dose assay whereas mosquitoes from site B may have been just under the threshold with 85 % mortality. This does not however mean that resistance is less of a threat in site B. The 15 % that did survive may have an extremely high level of resistance enabling them to readily survive long periods of time on a treated surface and thus potentially transmit malaria despite high coverage with vector control. In contrast, if the 50 % that survived the discriminating dose in site A have a relatively weak phenotype they will be killed when exposed to field concentrations of insecticide and thus pose less of an immediate threat. Data from Burkina Faso further illustrate how simply collating data on the prevalence of resistance can mask important changes in the strength of this resistance. Three years of monitoring insecticide resistance in *An. gambiae* from Vallée du Kou, in Southwest Burkina Faso using discriminating dose assays showed no significant difference in percentage mortality between the years but when a more quantitative measure was used to assess the strength of this phenotype, resistance was found to have increased ten -fold in a single year [12].

With no new insecticides expected to be licensed for use in malaria control before the end of the decade at the earliest, programmes need to make difficult decisions when faced with growing reports of resistance. Ideally a resistance management programme would be proactive rather than reactive but with only one insecticide class licensed for use on bed nets, and alternatives to pyrethroids frequently incurring higher costs for IRS, in reality, evidence of control failure is likely to be the only trigger for a change in insecticide policy. However, rather than waiting for insecticide failure to result in more deaths, it must be possible to re-define the way in which resistance is measured in the field to identify an ‘operationally significant’ threshold of resistance above which the gains from use of this insecticide class are lost. A necessary first step in this process is the development of simple bioassays that can measure resistance intensity so that resistance can be stratified according to the threat of control failure.

In this study, a variety of quantitative bioassays were used to assess the level of resistance in two laboratory strains and a field population of *An. gambiae s.l*. The consistency between the different assays and the relative ease of performing each method in the field were compared and the requirements for a reliable method that could be readily adopted under field conditions are discussed.

## Methods

### Mosquito strains

Two pyrethroid resistant laboratory strains of *An. gambiae s.l.* were used in the study with data from the insecticide susceptible Kisumu strain being used as a comparator. The Tiassalé strain was colonized from Southern Côte d’Ivoire in 2013 and maintained at the Liverpool School of Tropical Medicine (LSTM) under six-monthly selection pressure with deltamethrin. This strain, which contains both *An. gambiae s.s.* and *Anopheles coluzzii*, is resistant to all four classes of insecticide currently available for malaria control [13]. The Tororo strain of *An. gambiae s.s*. was colonized from Eastern Uganda in 2013 and maintained at LSTM without selection pressure. In addition to the pyrethroid resistance described in this report, this strain is resistant to bendiocarb (65 % mortality after 1 h exposure to 0.1 % papers) and DDT (8 % mortality after exposure to 4 % papers).

Bioassays on wild caught mosquitoes were performed between May and September, 2014, on *An. gambiae* adults raised from larval collections from Tiefora, Banfora District, Burkina Faso (GPS coordinates: 10;37;54.02, 04;33;22.85). Both *An. gambiae s.s*. and *An. coluzzii* are found sympatrically in this site with *An. gambiae* predominating (63 % *n* = 168, June-September 2014).

All bioassays were performed on 3–5 days old, non blood-fed females. Bioassays on the laboratory colonies were performed in the insectaries at LSTM. Assays on the Tiefora population were performed at the Centre National de Recherche et de Formation sur le Paludisme (CNRFP) insectaries in Banfora. In all bioassays mosquitoes were considered dead when they couldn’t stand or fly in a coordinated way.

### WHO susceptibility assays

WHO susceptibility tests were performed using papers obtained from Universiti Sains Malaysia, impregnated with 0.75 % permethrin. Exposure time was one hour and mortality was recorded 24 h later. Approximately 100 mosquitoes (four replicates of 25 mosquitoes) were used per test and the average mortality and the binomial confidence interval (95 %) calculated.

WHO susceptibility assays were also used to generate time response data. The standard 0.75 % papers were used but exposure time was varied from 5 min to 20 h (minimum of five time points per strain). The mean mortality was recorded per time point and the LT_50_ estimated using the Dose Effect function on XLSTAT (Microsoft).

### CDC bottle bioassays

A modified version of the published Centers for Disease Control and prevention (CDC) bottle bioassays was used to generate dose response data. Glass 250 ml bottles were coated with different concentration of permethrin ranging from 5 μg/ml to 200 μg/ml with between six and nine concentrations used per strain. Bottles were prepared according to CDC guidelines [14], but with a more stringent bottle washing process that involved rinsing them twice with acetone, washing with soap, rinsing with clean water and leaving them overnight in fresh water to eliminate any trace of soap. Approximately 25 mosquitoes were aspirated into the bottles for one hour and subsequently transferred to insecticide free paper cups, with a source of sugar solution, and mortality was recorded 24 h later. Four to six replicates were performed for each concentration with a control bottle (impregnated with acetone only) run alongside each insecticide concentration. Equivalent age mosquitoes from the Kisumu laboratory susceptible strain were exposed to insecticide concentrations ranging from 0.20 μg/ml to 5 μg/ml. The lethal concentration giving 50 % of mortality (LC_50_) was calculated as above.

### Resistance intensity rapid diagnostic test (I-RDT)

This is a simplified version of the CDC bottle bioassay described above in which fixed concentrations of insecticide are used. Four pre-measured vials containing permethrin which, when diluted in acetone and applied to 250 ml bottles give insecticide concentrations 1x, 2x, 5x and 10x (21.5 μg/ml, 43 μg/ml, 107.5 μg/ml and 215 μg/ml, respectively) the diagnostic dose were provided by CDC, Atlanta. These dosages for permethrin are those recommended in the CDC resistance intensity rapid diagnostic test (I-RDT) protocol now included as an insert in the 2010 CDC bottle bioassay manual [14]. Four replicates of 500 μl of acetone were added to each insecticide vial, and then transferred to a falcon tube and a further 48 ml of acetone added. The insecticide solutions were stored at 4 °C in the dark until use. 1 ml of insecticide solution was applied to 250 ml glass bottles and mosquitoes exposed for 30 min as described in the CDC bottle bioassay manual. At the end of the exposure the mosquitoes were transferred to insecticide free paper cups and immediate knockdown was recorded. The mosquitoes were provided with sugar solution, and retained in the paper cups in the insectary for a further 24 h before mortality was recorded.

### Cone bioassays

Cone bioassays were performed using Olyset and Permanet 2.0 nets, provided directly by the manufacturer (Sumitomo Chemical Ltd and Vestergaard, respectively). Ten replicates of ten mosquitoes were tested on net pieces selected randomly from the nets. Five replicates were exposed to an untreated net as control experiments. Mosquitoes were exposed for 3 min and the 60 min knock-down and 24 h morality recorded. Significant differences between knockdown or mortality between strains were determined by pairwise comparisons using the z-test and the software programme VassarStats.

## Results

### WHO susceptibility assays

**Fig. 1:**
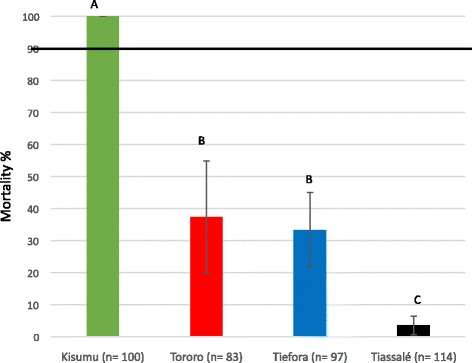
Discriminating dose. Mortality rates after exposure to the WHO discriminating dose of permethrin. Error bars represent 95 % binomial confidence intervals. Columns with a different letter are significantly different to each other. The 90 % threshold used by WHO to define a resistant population is shown by a horizontal line

The percentage mortality after exposure to the WHO discriminating dose of permethrin (0.75 %) for 60 min varied from 100 % for Kisumu to 3.5 % for Tiassalé. There was no significant difference in mortality rates for Tororo and Tiefora, which both had less than 40 % mortality (Fig. 1).

**Fig. 2:**
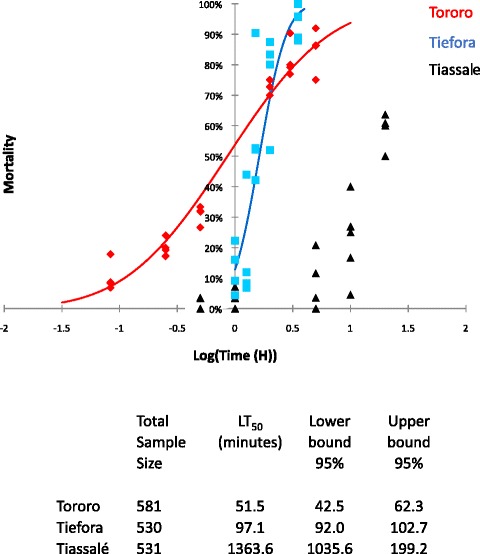
Time response. Time response curves for adult females exposed to 0.75 % permethrin in WHO susceptibility tube assays. Best fit lines are plotted using XLSTAT. Tiefora and Tororo were exposed for five or six different time points ranging from 5 to 300 min. Tiassalé was exposed four time points from 30 min to 20 h

Exposure time was then varied and the time mortality response plotted (Fig. 2). The time required to obtain 50 % mortality (LT_50_) was estimated to be 51.5 min (95 % confidence intervals (CIs) 42.5–62.3) and, 97.1 min (95 % CIs 92.0–102.7) for Tororo and Tiefora strains respectively. For Tiassalé the longest exposure time used in the experiment (20 h) only gave 58 % mortality although the best fit curve for the data gave estimated the LT_50_ to be over 22 h. The permethrin LT_50_ for Kisumu females has been previously determined as 7.8 min [15]. Using this Kisumu data as the denominator, the resistance ratios for the three strains according to the LT_50_ values are 6.6-fold for Tororo, 12.4-fold for Tiefora and 174.8-fold for Tiassalé.

### CDC bottle bioassays

**Fig. 3:**
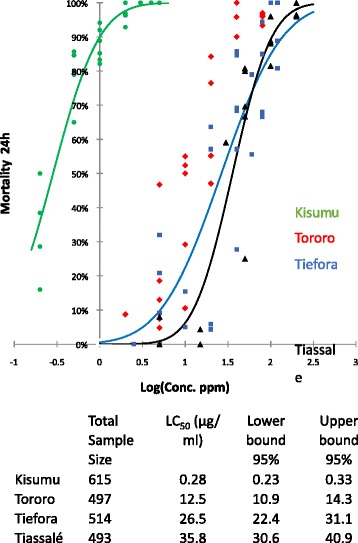
Dose response. Dose response curves for adult females exposed to permethrin for 60 min in CDC bottle bioassays. Best fit lines are plotted using XLSTAT. A minimum of six concentrations were used for each strain

Keeping a fixed exposure time of 60 min but varying the concentration of insecticides using the bottle bioassays enabled the permethrin concentration required to achieve 50 % mortality to be estimated (Fig. 3). For Tororo this was 12.5 μg/ml (95 % CI 10.9–14.3), for Tiefora 26.5 μg/ml (22.4–31.1) and for Tiassalé 35.8 μg/ml (30.6–40.9). By comparison the LC_50_ for the susceptible Kisumu strain was just 0.23 μg/ml (0.058–0.34) leading to resistance ratios of 54.3-fold, 115.2 -fold and 155.6-fold for Tororo, Tiefora and Tiassalé, respectively.

### Resistance Intensity Rapid Diagnostic Test (I-RDT)

**Fig. 4:**
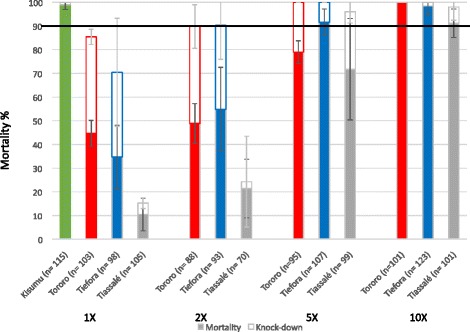
CDC resistance intensity rapid diagnostic test. Knockdown after a 30 min exposure (open box), and mortality after a 24 h recovery (solid box) to bottles coated with different multitudes of the CDC bottle bioassay diagnostic dose for permethrin. Error bars represent 95 % binomial confidence intervals. The 90 % threshold used by CDC to define a resistant population is shown by a horizontal line

An alternative approach to using the CDC bottle bioassays termed the Resistance Intensity Rapid Diagnostic Test (I-RDT) (14) was also evaluated. Pre-measured insecticide vials with different multitudes of the diagnostic dose were used to record the immediate knockdown at the end of the 30 min exposure and the 24 h mortality (Fig. 4). The CDC bottle bioassay guidelines recommend a cut off of less than 90 % knockdown at the end of the assay as the definition of a resistant population. Using this criteria, Kisumu was susceptible to the 1x diagnostic dose (21.5 μg/ml), the Tororo and Tiefora strain were resistant to 1x, but susceptible to the 2x diagnostic dose and Tiassalé was resistant to 1x and 2x but susceptible to 5x the diagnostic dose. However, when 24 h mortality was used at the end point, Kisumu was still susceptible to the 1x dose, but Tiefora was resistant to the 2x dose but susceptible to 5x and both the Tororo and Tiassalé strains were resistant to 5x the diagnostic dose. The highest concentration (215 μg/ml) resulted in almost complete knockdown in all three strains and >90 % mortality.

### Cone bioassays

**Fig. 5:**
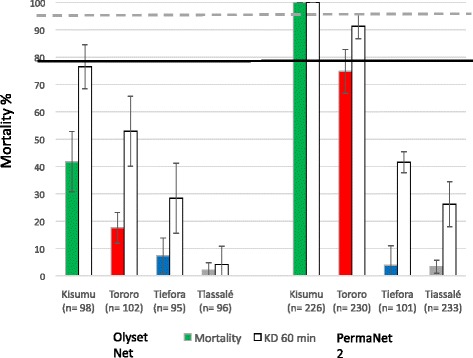
Cone bioassays. Knockdown after a 3-min exposure (open box), and mortality after a 24-h recovery (solid box) to long-lasting insecticide-treated bed nets using a cone bioassay. Error bars represent 95 % binomial confidence intervals. The solid line and the dotted line represents the 80 % mortality and 95 % knockdown threshold used by WHO to test the efficacy of LLINs in phase I studies

When mosquitoes were exposed to a new Olyset Net LLIN, which has permethrin incorporated into the polyethylene fibres, mortality rates less than 50 % were observed for all strains, including the Kisumu susceptible strain (Fig. 5). Exposure to PermaNet 2.0, whose polyester fibres are coated with deltamethrin, resulted in 100 % mortality in the susceptible laboratory strain but with less than 80 % mortality in all three resistant strains (Fig. 5). For both nets mortality rates were lowest for the Tiassalé strain, followed by Tiefora and highest mortality was seen for Tororo.

## Discussion

In 2012, the WHO published the Global Plan for Insecticide Resistance Management in malaria vectors (GPIRM) in response to the growing number of countries reporting insecticide resistance in Anopheles mosquitoes [1]. This document provides information on current monitoring guidelines and outlines alternative resistance management strategies that countries can adopt to mitigate, or preferably delay, the onset of insecticide resistance. However, all substitutes to pyrethroids for IRS are more expensive and the only alternative to pyrethroid-treated LLINs currently available are nets containing pyrethroid and PBO, which, again, have a higher unit cost. Therefore, malaria control programmes face challenging decisions when insecticide resistance emerges. A textbook insecticide resistance management strategy requires a change in insecticide class as soon as there is any sign of resistance in a population. But in reality, indications of control failure with insecticide are likely to be the trigger for a change in insecticide use. An indication of the threshold at which resistance is likely to negatively impact on control would aid decision making by providing pragmatic guidance on when it is necessary to respond. The GPIRM document does recommend alternative strategies depending on the resistance mechanism(s) involved, but mechanistic studies are not always feasible in settings with constraints on available expertise and resources. To define this threshold of ‘operationally significant resistance’, it is necessary to agree on a standardized method for quantifying the strength of resistance that can supplement information on resistance prevalence that is already being routinely generated.

The current study compared results obtained using different bioassay methodologies. Three populations were investigated that were all classified as resistant according to the current WHO definition (Fig. 1) plus a laboratory susceptible strain as a comparator. Two of the resistant populations were a mixture of two *An. gambiae* complex species*, An. gambiae s.s.* and *An. coluzzii*. For the Tiefora population, in which *An. gambiae* was the major vector, the study verified that there was no significant difference in the species composition between the general population and those surviving the 2 x intensity assay (*p* = 0.757). Tiassalé has been maintained in colony for multiple generations and has a large proportion of hybrids. Both single species and hybrids survive the diagnostic dose. The presence of mixed populations could be seen as a weakness of the study but does reflect the reality of many field studies, where species ID is not possible.

The LT_50_ and LC_50_ measurements both ranked the strength of resistance as highest in Tiassalé, followed by Tiefora and lastly Tororo although the confidence intervals for the LC_50_ for Tiefora and Tiassalé overlap. Using the I-RDT and 24 h mortality as an end point, Tiassalé and Tororo both fall into the same category with Tiefora showing a lower level of resistance but when knockdown is used, Tiassalé is categorized as being in a higher resistance class than either Tororo or Tiefora.

The two fully quantitative assays proved challenging at different ends of the resistance spectrum. Reliable measurements for the LT_50_ could be obtained for susceptible strains and populations with relatively low levels of resistance but this methodology was not well suited for the Tiassalé strain as, even at the maximum exposure time (20 h) high levels of mortality were not obtained (Fig. 2). Such high exposure times may result in mortality that is due to factors other than the insecticide itself, although in this study, the mortality in the control tube after 20 h was zero (*n* = 52). This method was very easy to perform in the field.

For the LC_50_ calculations, difficulties arose at the other end of the spectrum. Here it proved difficult to obtain a reliable value for the susceptible Kisumu strain with the lowest concentration giving 33 % mortality (Fig. 3). The problem is even more acute for deltamethrin where the quantities of insecticide that need to be measured are an order of magnitude lower. Measuring resistance strength using the bottle bioassay method was more challenging to perform under field settings as it required access to technical grade insecticide and a fine scale balance. The stringency of the bottle washing procedure also needed to be increased to avoid cross contamination issues.

Despite these challenges, the permethrin resistance ratio calculated for the Tiassalé strain, using Kisumu as the denominator, was comparable for the two methods (175-fold using the LT_50_; 156-fold using the LC_50_). However wider discrepancies were seen for the measurements for the less resistant Tororo and Tiefora strains where LC_50_ RR estimates were 8–9 × higher than LT_50_). There is very little published data on resistance strength to compare these data sets to and certainly no studies that have compared resistance ratios obtained using LC and LT data. Resistance ratios of 138 and 292 –fold, calculated using LT_50_ measurements, have been reported in field populations of *An. gambiae s.l*. exposed to deltamethrin in Côte d’Ivoire and Uganda, respectively [13, 15], and a recent study in Zambia (recording knockdown rather than mortality) found time to knockdown with deltamethrin was approximately 14-fold higher in a wild population of *An. funestus* than in the laboratory susceptible strain [16]. The highest resistance ratios reported to date are from Burkina Faso where, in 2012, *An. coluzzii* populations were 650 × more resistant to deltamethrin than the Kisumu strain using LT measurements. The following year, resistance ratios >1000 were reported from the same study site using bottle bioassays and comparing LC50s [12].

The interpretation of the intensity assays results was heavily dependent on whether immediate knockdown (KD) at the end of the assay or 24-h mortality was used as the metric. Using KD, the Tororo strain is borderline susceptible (90 % knockdown). Furthermore, all three strains would be classed in a lower resistance category using KD than mortality. Differences in the outcomes from the two metrics likely reflect the role of different resistance mechanisms. For populations where knockdown resistance or *kdr* is the major contributing resistance mechanism, KD rates might be expected to be lower than mortality, as the target site mutation enables mosquitoes to temporarily withstand pyrethroid exposure. Whereas for metabolic mechanisms, or when the insecticide exposure exceeds the protection from KD afforded by the *kdr* allele, mortality rates may be lower than KD as knocked down mosquitoes are able to detoxify the insecticide rapidly enough to recover after exposure is removed. A primary consideration in selecting the end point to record should be the value of the output to decision making in insecticide use: is a mosquito that survives knockdown or a mosquito that is temporarily intoxicated but later recovers the greatest threat? The answer to this may be dependent on the mode of application of the insecticide with a mortality being of more relevance for measuring the efficacy of IRS applications and both KD and mortality being of value for assessing the insecticidal activity of pyrethroid-treated nets.

Reaching a consensus on how to measure the strength or intensity of resistance is only the first step. Agreement is also needed on what level of resistance has an operational impact. In the agricultural sector, definitions of operationally significant resistance are often related to the field dose of insecticide by dividing the LC_50_ by the field dose [17]. However this is not so straightforward in vector control as formulation issues can have a major impact on the bioavailability of insecticide making the field dose difficult to determine.

Although responses to the field dose were not quantified in this study, cone bioassays were included to compare how the three populations responded to the bioavailable insecticide on the surface of a LLIN. Using PermaNet 2.0, only the susceptible Kisumu strain met the WHO criteria of > 95 % knockdown and > 80 % mortality. Mortality rates with Tiefora and Tiassalé were below 10 % suggesting that the performance of these nets against these populations would be severely compromised. Results from the Olyset nets were difficult to interpret as the mortality and knockdown rates were below WHO criteria even for the susceptible strain. Previous studies have also reported that Olyset nets perform poorly in cone bioassays [18]. However, it is noted that the mortality with the three resistant strains was significantly lower than the susceptible strain, indicating that resistance is also having an impact on efficacy of this net type. Tunnel bioassays [19] have been proposed as a more realistic measure of the performance of the different net types but these are less amenable to routine monitoring programmes where obtaining the necessary equipment and live animals may be problematic.

An alternative approach to defining an operationally significant resistance threshold would be to utilize data from experimental hut studies. In principle data on the performance of nets could be correlated with the strength of resistance. Whilst this could be done using resistance prevalence data from discriminating dose assays, for reasons outlined above, introducing a more quantitative measure of resistance would improve the rigor of this analysis.

Finally, it should be noted that the source of mosquitoes used in the bioassays could have a considerable impact on the data. It is well documented that age, physiological status and larval rearing environment affects the resistance status of adults [20–22]. In this study the bioassays on field populations were all performed on adults raised form larvae to standardize between assays but this is not always practical, nor indeed desirable. It could be argued that the information of most value to control programmes is the resistance level of the entire population of potential vectors which would argue for bioassays directly on indoor (or outdoor) adult collections. As usual, the best approach depends on the question being asked. If comparing response between sites, or over time is the goal then some element of standardization on which mosquitoes are tested is necessary but if a quick assessment of the probably resistance status in a location is what is required, the use of wild caught adults may suffice.

## Conclusion

The objective of this study was to compare the results, and practicalities, of using alternative measures to quantify the level of resistance to a single insecticide in different populations. Each method has its own merits and disadvantages and there were notable differences in the results obtained from each bioassay. Of the currently available assays, the intensity assays are perhaps the best compromise between ease of performance and data richness but further validation of these assays, and guidelines on data interpretation is still needed. Given the widespread acceptability of the current discriminating dose assays from WHO, consideration should be given to the centralized production of standardized papers impregnated with a range of insecticide concentrations to enable resistance intensity to be estimated. Finally, the value of measuring resistance intensity is dependent on the ability to extrapolate from this data to predict the performance of insecticide based vector control tools in different resistance settings. In this regard, it is recommended that cone bioassays be used to assess the response of local field mosquitoes to the field dose and formulation of insecticide being used and, where possible, controls using susceptible mosquitoes should also be performed. In addition, attempts to correlate resistance strength in *Anopheles* mosquitoes with epidemiological indicators of malaria should be intensified.
